# An Effectual Biosorbent Substance for Removal of Manganese Ions from Aquatic Environment: A Promising Environmental Remediation Study with Activated Coastal Waste of *Zostera marina* Plant

**DOI:** 10.1155/2020/7806154

**Published:** 2020-07-07

**Authors:** Fatih Deniz, Elif Tezel Ersanli

**Affiliations:** ^1^Department of Environmental Protection Technologies, Bozova Vocational School, Harran University, 63850 Bozova/Sanliurfa, Turkey; ^2^Department of Biology, Faculty of Arts and Science, Sinop University, 57000 Sinop, Turkey

## Abstract

In the present research paper, a biosorptive remediation practice for an aqueous medium sample polluted with manganese ions was implemented using the activated coastal waste of the *Zostera marina* plant. This is the first report in the literature on the utilization of current modified biological waste as a biosorbent substance for the removal of manganese ions from the water environment. The analyses of biosorbent characterization, environmental condition, kinetic, equilibrium, and comparison were performed to introduce the ability of prepared biosorbent for the removal of manganese from the aquatic medium. The biosorbent matter has a rough surface with numerous cavities and cracks and various functional groups for the biosorption of manganese. The environmental conditions significantly affected the manganese purification process, and the optimum working conditions were determined to be biosorbent quantity of 10 mg, pH of 6, manganese concentration of 30 mg L^−1^, and time of 60 min. The pseudo-second-order model best explained the kinetic data of biosorption operation. The biosorption equilibrium data were best described by the Freundlich isotherm. According to the Langmuir equilibrium model, the maximum purification potency was estimated to be 120.6 mg g^−1^. The comparison work revealed that the activated coastal waste of the *Z*. *marina* plant could be utilized as an effectual and promising biosorbent substance for the remediation of an aquatic environment contaminated with manganese ions.

## 1. Introduction

Today, heavy metal pollution is a leading environmental trouble worldwide because of the severe noxious effects of heavy metal ions to human beings and natural ecosystems [1]. These substances are widely used in different industrial activities like plating, smelting, tanning, mining, plastic, alloy, electronic, paint, tire, fertilizer, battery, ceramic, and agrochemical production processes. After use, considerable amounts of heavy metal ions are frequently released into the receiving environment, especially the water environment without proper clarification [2, 3]. These metallic elements are toxic, carcinogenic, accumulative, stubborn, and nondegradable [1, 4]. Manganese, one of the metals naturally found on Earth, is an essential micrometallic element for plants, animals, and human beings. The excessive increase in various industrial operations has enhanced the manganese concentration in the receiving water environment. According to the World Health Organization (WHO), the maximum acceptable concentration of manganese in drinking water is 0.05 mg L^−1^. The high concentration of manganese causes various physiological, biochemical, and morphological problems in living beings. Manganese is also included in the Hazardous Substances Priority List by the Toxic Substances and Disease Registry (ATSDR) [5, 6]. Therefore, the removal of extra manganese ions from the water environment is extremely important to extinguish the noxious effects on humans and other living things.

Biosorption among environmental remediation practices is a leading-edge refinement technique for the heavy metal pollution in an aqueous environment by virtue of its low cost, high performance, easy design, simple operation, and ecofriendly features [3, 7]. The activated carbon is the top sorbent matter utilized in biosorption practice owing to great physicochemical structural properties. However, the problems like high manufacturing expense and low regeneration capacity restrain the influential use of activated carbon in the large-scale heavy metal decontamination applications [8, 9]. In recent years, due to their properties such as high abundance, biodegradable, low cost, renewable, high regeneration capacity, environmentally friendly, and high yield, the utilization of natural biological wastes instead of activated carbon as a sorbent matter has become an attractive area of research [10, 11].

In this study, the coastal waste of the *Zostera marina* plant was used to create an effectual biosorbent substance for the removal of manganese ions from the aquatic environment. This coastal plant waste is produced annually in great quantities. The conversion of this biological waste to an effectual biosorbent substance for the environmental remediation practices not only reduces the ecological risk imposed by dumping it into the natural environment but also eliminates the cost of waste management [9, 12]. On the other hand, an activation operation is frequently performed to increase the biosorption capacity of the biosorbent and to prevent the release of soluble organic compounds into the water environment. The soluble organic compounds cause a secondary pollution in the aquatic environment owing to increases in the biological/chemical oxygen demand and total organic carbon amount [9, 11]. Thus, the coastal waste of the *Z. marina* plant was modified with dilute sodium hydroxide solution known as a successful activation agent. The analyses of characterization, environmental condition, kinetic, equilibrium, and comparison were performed to introduce the ability of the prepared biosorbent for the removal of manganese ions from the aquatic medium. According to our comprehensive literature research, there is no report on the utilization of current modified biological waste as a biosorbent substance for the biosorption of manganese ions from the water environment.

## 2. Materials and Methods

### 2.1. Activated Biosorbent Substance Preparation and Characterization

The coastal waste of the *Z. marina* plant was supplied from the region of Black Sea (Sinop, Turkey). The plant waste was washed several times with normal water and then distilled water to remove impurities. It was dried in an oven at 80°C until constant weight. The dried plant waste was grounded using a laboratory grinder and sieved to obtain the particles having a size of less than 0.5 mm. Then, a sample of 1 g of obtained material was slowly stirred with 100 mL of sodium hydroxide solution (0.3 mol L^−1^) (Merck, Germany) during 24 hours at ambient temperature for the activation operation. It was again washed several times with distilled water to remove excess chemical substance and dried in an oven at 80°C until constant steady weight. The modified biosorbent substance was stored in an airtight glass cover for the removal of manganese ions from the aquatic environment. The surface features of the activated biosorbent before and after the biosorption process were characterized using a scanning electron microscope (SEM, Zeiss, Germany). The functional group profiles of the original and manganese-loaded biosorbent samples were analyzed by a Fourier-transform infrared spectrometer (FTIR, PerkinElmer, USA) in the wavenumber range of 4000-400 cm^−1^.

### 2.2. Manganese Solution Preparation

Manganese (II) chloride dehydrate chemical was provided from Merck, Germany, and utilized as a model heavy metal item to evaluate the environmental remediation potential of a biosorbent substance. A stock manganese solution was prepared at a concentration of 1000 ppm using distilled water, and the desired concentrations were freshly prepared from the stock by the dilution method. The initial pH values of the prepared solution samples were adjusted using the chemical solutions of 0.1 mol L^−1^ of hydrochloric acid (Merck, Germany) and 0.1 mol L^−1^ of sodium hydroxide (Merck, Germany). These chemicals and also other chemicals used in this study were of analytical grade.

### 2.3. Batch Manganese Removal Experiment

The biosorptive manganese removal study was conducted with a series of Erlenmeyer flasks containing 100 mL of known concentration of manganese solution with a given amount of the biosorbent material on an orbital shaker at 150 rpm and 24°C. The effects of the environmental conditions such as the biosorbent quantity (*M*, 10-30 mg), pH (pH, 2-6), manganese concentration (*C*_0_, 10-30 mg L^−1^), and time (*t*, 0-120 min) on the manganese removal potential of the biosorbent from the aqueous medium were studied using the one-variable-at-a-time (OVAT) experiment method. At the end of the desired operation time, a known volume of experiment solution was removed from the Erlenmeyer flask and subjected to the separation process. The residual concentration of manganese in the experiment medium was determined using a test kit set of manganese ion (catalogue number: 114770, Spectroquant, Merck, Germany) by a UV–visible spectrophotometer (445 nm). The biosorption work was carried out twice and the mean result of the experiment was used to determine the biosorption capacity of the biosorbent, *q*_t,e_ (mg g^−1^), by the equation below:

(1) $$\begin{array}{c} q_{\text{t},\text{e}}=\frac{\left(C_{0}-C_{\text{t},\text{e}}\right)V}{M}, \end{array}$$

where *C*_0_ is the initial manganese concentration (mg L^−1^), *C*_t,e_ is the manganese concentration at a time *t* or at equilibrium (mg L^−1^), *V* is the volume of aqueous medium (L), and *M* is the weight of the biosorbent (g).

### 2.4. Kinetic Modeling Study

The kinetic modeling study of manganese removal from the water environment was performed using the pseudo-first-order [13], pseudo-second-order [14], Elovich [15], and intraparticle diffusion [16] models:

(2) $$\begin{array}{c} \text{Pseudo}-\text{first}-\text{order}:q_{\text{t}}=q_{\text{e}}\left(1-{\text{e}}^{-k_{1}t}\right), \end{array}$$

where *k*_1_ is the rate constant (min^−1^) and *q*_e_ is the equilibrium capacity of the biosorbent (mg g^−1^).

(3) $$\begin{array}{c} \text{Pseudo}-\text{second}-\text{order}:q_{\text{t}}=\frac{k_{2}{q_{\text{e}}}^{2}t}{1+k_{2}q_{\text{e}}t}, \end{array}$$

where *k*_2_ is the rate constant (g mg^−1^ min^−1^), and *q*_e_ is the equilibrium capacity of the biosorbent (mg g^−1^).

(4) $$\begin{array}{c} \text{Elovich}:q_{\text{t}}=\frac{1}{\beta }\ln\left(1+\alpha \beta t\right), \end{array}$$

where *α* is the initial rate (mg g^−1^ min^−1^) and *β* is the desorption constant (g mg^−1^).

(5) $$\begin{array}{c} \text{Intraparticle diffusion}:q_{\text{t}}=k_{\text{p}}t^{1/2}+C, \end{array}$$

where *k*_p_ is the rate constant (mg g^−1^ min^-1/2^) and *C* is the intercept (mg g^−1^). The values of the characteristic parameters of kinetic models were determined using the nonlinear regression method by SigmaPlot software (Systat, USA). The determination coefficient (*R*^2^) and standard error (SE) analyses were utilized to assign the best models describing the biosorption kinetic data.

### 2.5. Equilibrium Modeling Work

Langmuir [17], Freundlich [18], and (Dubinin-Radushkevich [19] isotherm equations were used to model the manganese biosorption equilibrium:

(6) $$\begin{array}{c} \text{Langmuir}:q_{\text{e}}=\frac{q_{\text{m}}K_{\text{L}}C_{\text{e}}}{1+K_{\text{L}}C_{\text{e}}},\\ R_{\text{L}}=\frac{1}{1+K_{\text{L}}C_{0}}, \end{array}$$

where *q*_m_ is the maximum capacity of the biosorbent (mg g^−1^), *K*_L_ is a constant related to the energy of biosorption (L mg^−1^), and *R*_L_ is the separation factor.

(7) $$\begin{array}{c} \text{Freundlich}:q_{\text{e}}=K_{\text{F}}{C_{\text{e}}}^{1/n_{\text{F}}}, \end{array}$$

where *K*_F_ is the biosorption capacity of the biosorbent (mg g^−1^ (L mg^−1^)^1/*n*^_F_) and *n*_F_ is the biosorption intensity.

(8) $$\begin{array}{c} \text{Dubinin}-\text{Radushkevich}:q_{\text{e}}=q_{\text{m}}{\text{e}}^{-B\varepsilon^{2}},\\ E=\frac{1}{{\left(2B\right)}^{1/2}}, \end{array}$$

where *q*_m_ is the maximum capacity of the biosorbent (mg g^−1^), *B* is a constant related to the biosorption energy (mol^2^ kJ^−2^), *ε* is the Polanyi potential (RT ln (1 + (1/*Ce*))), *R* is the gas constant (J mol^−1^ K^−1^), *T* is the temperature (K), and *E* is the mean free energy of biosorption (kJ mol^−1^). The values of the characteristic parameters of the isotherm models were determined using the nonlinear regression method by SigmaPlot software. The analyses of *R*^2^ and SE were utilized to assign the best models describing the biosorption equilibrium data.

## 3. Results and Discussion

### 3.1. Features of Biosorbent Material

**Figure 1:**
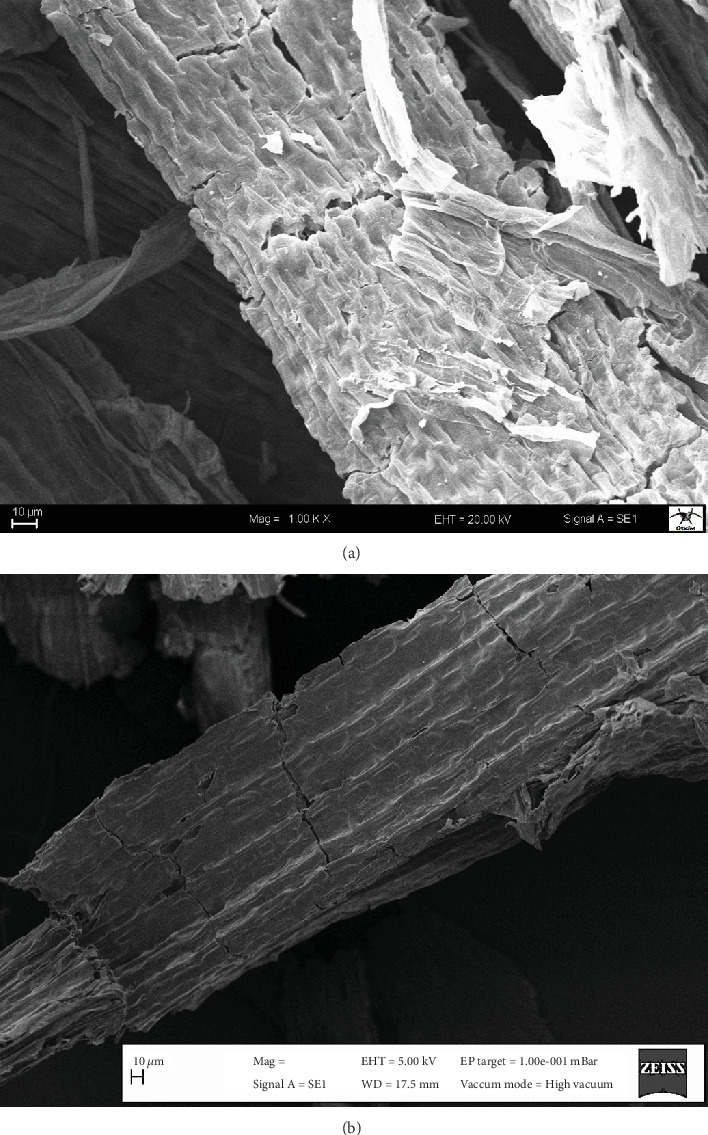
SEM images of activated biosorbent before (a) and after (b) manganese biosorption.

The surface properties of the modified biosorbent before and after the manganese biosorption process are presented in Figure 1. The biosorbent has a rough surface with numerous cavities and cracks. Such surface features provide a large and effective manganese bonding area to the biosorbent substance [20]. The morphological changes after the manganese biosorption process showed that the surface of the biosorbent was clogged by the manganese ions.

**Figure 2:**
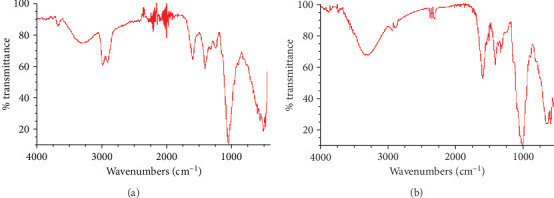
FTIR profiles of original (a) and manganese-loaded (b) biosorbent samples.

The functional group profiles of the original and manganese-loaded biosorbent samples are indicated in Figure 2. The bands at around 3650 cm^−1^ and 3300 cm^−1^ are associated with the O-H and N-H groups. The bands seen in the range of 2700-3000 cm^−1^ refer to the group of C-H [21]. The band at about 1600 cm^−1^ is related to the C=O or C=C groups [22]. The bands observed in the range of 1200-1500 cm^−1^ denote the groups of C-N and C-O [9, 23]. The sharp band that appeared at around 1050 cm^−1^ is assigned to the C-O-C group [22]. The bands in the range of 750-900 cm^−1^ are attributed to the C-H group [24]. After the manganese biosorption, a lot of characteristic bands of biosorbent shifted, appeared, or disappeared. These changes showed that various active groups were involved in the manganese removal from the water environment by the biosorbent.

### 3.2. Effect of Biosorbent Quantity

**Figure 3:**
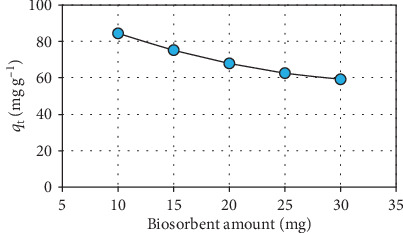
Effect of biosorbent amount.

Figure 3 shows the effect of biosorbent amount on the manganese biosorption (*M*: 10-30 mg; *C*_0_: 25 mg L^−1^; *t*: 120 min; and pH: 6). This environmental parameter decreased the manganese removal capacity of the biosorbent. At higher biosorbent amounts, this decreasing trend observed in the biosorption activity of the biosorbent substance can be attributed to the presence of unsaturated binding sites and the decline in the effective surface area of the biosorbent for the biosorption of manganese ions [25, 26].

### 3.3. Effect of pH

**Figure 4:**
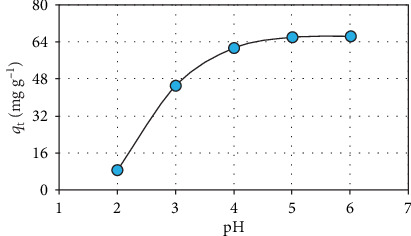
Effect of pH.

Figure 4 indicates the effect of pH on the manganese removal from the water medium by the biosorbent (pH: 2-6; *C*_0_: 15 mg L^−1^; *t*: 120 min; and *M*: 10 mg). A significant increase in the biosorption activity was observed as the environmental pH increased. The manganese biosorption capacity of the biosorbent decreases under the high acidic environment conditions because of the repulsive forces between the positively charged functional groups and the metal cations. On the contrary, the biosorption capacity increases under the low acidic environment conditions owing to the attractive forces between the negatively charged functional groups and the metal cations [27, 28].

### 3.4. Effects of Concentration of Manganese and Time of Removal Operation

**Figure 5:**
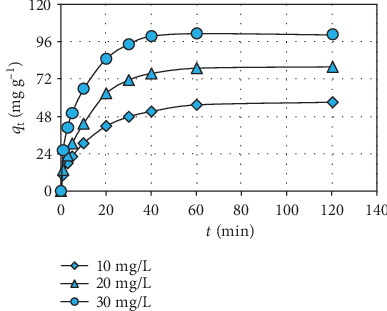
Effect of manganese concentration and biosorption operation time.

Figure 5 displays the effect of manganese concentration on the biosorption activity of the biosorbent (*C*_0_: 10-30 mg L^−1^; *t*: 0-120 min; pH: 6; and *M*: 10 mg). The increase in the manganese concentration enhanced the biosorption potential of the biosorbent. This increase tendency in the biosorption yield at higher manganese charge in the aqueous medium is attributed to the higher concentration gradient generating a greater driving force for the mass transfer process between the biosorbent and aquatic environment [22, 29].

Figure 5 also demonstrates the effect of biosorption operation time on the capacity of the biosorbent (*t*: 0-120 min; *C*_0_: 10-30 mg L^−1^; pH: 6; and *M*: 10 mg). The biosorption efficiency of the biosorbent increased rapidly in the early phases of biosorption operation owing to the presence of numerous free binding places on the biosorbent surface and high manganese concentration difference between the biosorbent and water environment [11, 30]. The biosorption capacity of the biosorbent increased weakly in the later phases of biosorption operation due to the gradual occupation of binding areas by the manganese ions and nearly attained an equilibrium value within 60 min.

### 3.5. Study of Biosorption Kinetic

**Figure 6:**
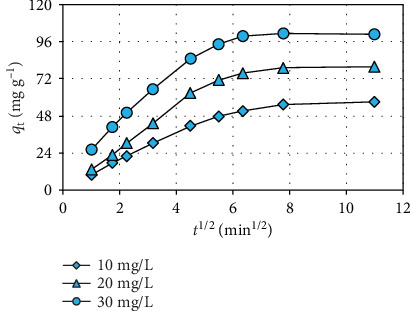
Graphs of intraparticle diffusion model for different manganese concentrations.

The kinetic modeling study of manganese removal from the water environment was achieved using the pseudo-first-order, pseudo-second-order, Elovich, and intraparticle diffusion models. The modeling results obtained are shown in Table 1. According to *R*^2^ and SE statistical analysis results, the pseudo-second-order model best described the kinetic data of biosorption. The pseudo-second-order model suggests that the biosorption of manganese from the aqueous medium is governed by a chemical kinetic mechanism involving electron exchange or sharing [26]. For the evaluation of the effect of diffusional behavior on the manganese biosorption mechanism, the graphs of the intraparticle diffusion model for the different manganese concentrations are given in Figure 6. The curves of the intraparticle diffusion model are not straight lines passing through the origin point. This event shows that the intraparticle diffusion was not the only control phase in the biosorption mechanism of manganese from the aqueous environment [31].

### 3.6. Study of Biosorption Equilibrium

Langmuir, Freundlich, and Dubinin-Radushkevich isotherm equations were used to model the manganese biosorption equilibrium. The modeling results are displayed in Table 2. The values of *R*^2^ and SE showed that the Freundlich isotherm was the best model explaining the equilibrium data of manganese biosorption. The Freundlich model suggests that the biosorption of manganese ions occurs as a multilayer on the heterogeneous surface of the biosorbent. Also, the *n*_F_ parameter of the Freundlich isotherm in the range of 1 to 10 indicates a favorable biosorption process [32]. The values of the *R*_L_ parameter of the Langmuir model in the range of 0 to 1 confirm the favorable biosorption [30]. The parameter of *E* from the Dubinin-Radushkevich isotherm less than 8 kJ mol^−1^ shows a physical biosorption process [33].

Table 3 presents the purge efficiencies of different sorbent materials for the manganese pollution. The maximum purification potency of the biosorbent used in this study was estimated to be 120.6 mg g^−1^ according to the Langmuir equilibrium model. This outstanding remediation potential indicated that the current activated biological substance could be used as a promising biosorbent for the removal of manganese ions from the aquatic environment. However, it should be noted that these manganese biosorption studies have different operating conditions as seen in the table. For a more accurate comparison, the biosorption studies should be performed under the same working conditions.

## 4. Conclusions

In the current work, a biosorptive manganese remediation operation was successfully implemented using the modified coastal waste of the *Z*. *marina* plant. The biosorbent has a rough surface with various functional groups. The working conditions significantly affected the manganese biosorption process. The pseudo-second-order and Freundlich models best explained the biosorption process. The maximum biosorption potency was estimated to be 120.6 mg g^−1^. The present study showed that this modified plant waste could be employed as an effectual and promising biosorbent for the biosorption of manganese from the water environment.

## Figures and Tables

**Table 1:** Biosorption kinetic modeling study.

Model	Parameter	Value	*R* ^2^	SE
Pseudo-first-order	*k*_1_ *q*_e_	0.141 (min^−1^) 97.885 (mg g^−1^)	0.9672	6.905
Pseudo-second-order	*k*_2_ *q*_e_	0.001785 (g mg^−1^ min^−1^) 108.508 (mg g^−1^)	0.9866	4.423
Elovich	*α* *β*	18.294 (mg g^−1^ min^−1^) 0.055 (g mg^−1^)	0.9722	6.360
Intraparticle diffusion	*k*_p_ *C*	26.078 (mg g^−1^ min^-1/2^) 9.416 (mg g^−1^)	0.7863	17.631

**Table 2:** Biosorption equilibrium modeling study.

Model	Parameter	Value	*R* ^2^	SE
Langmuir	*q*_m_ *R*_L_	120.6 (mg g^−1^) 0.140-0.328	0.9361	7.794
Freundlich	*K*_F_ *n*_F_	33.536(mg g^−1^ (L mg^−1^)^1/*n*^_F_) 2.756	0.9833	3.984
Dubinin-Radushkevich	*q*_m_ *E*	95.464 (mg g^−1^) 0.526 (kJ mol^−1^)	0.8480	12.022

**Table 3:** Purge efficiencies of different sorbent materials for manganese pollution.

Material	Purification potency (mg g^−1^)	Conditions	Reference
Activated coastal waste of *Z*. *marina*	120.6	*M*: 0.1 g L^−1^; *C*_0_: 30 mg L^−1^; *t*: 60 min; pH: 6	This study
Granular activated carbon	7.63	*M*: 0.2 g L^−1^; *C*_0_: 2 mg L^−1^; *t*: 30 min; pH: 7	[34]
Modified acorn of *Quercus ithaburensis*	12.1359	*M*: 5 g L^−1^; *C*_0_: 30 mg L^−1^; *t*: 60 min; pH: 4.6	[35]
*Pseudomonas* sp.	109	*M*: 1 g L^−1^; *C*_0_: 200 mg L^−1^; *t*: 10 min; pH: 6	[36]
Surfactant-modified alumina	1.30	*M*: 20 g L^−1^; *C*_0_: 70 mg L^−1^; *t*: 30 min; pH: 6	[37]
Functionalized mesoporous silica	88.9	*M*: 0.5 g L^−1^; *C*_0_: 200 mg L^−1^; *t*: 40 min; pH: 7	[38]
*Blakeslea trispora*	40	*M*: 1 g L^−1^; *C*_0_: 200 mg L^−1^; *t*: 150 min; pH: 6	[36]
Fe_3_O_4_ nanoparticles	64.27	*M*: 2 g L^−1^; *C*_0_: 100 mg L^−1^; *t*: 24 h; pH: 6	[39]
Shell of *Portunus sanguinolentus*	69.9	*M*: 5 g L^−1^; *C*_0_: 500 mg L^−1^; *t*: 8 h; pH: 6	[40]
PVA/chitosan nanoparticles	10.515	*M*: 8 g L^−1^; *C*_0_: 100 mg L^−1^; *t*: 90 min; pH: 5	[41]
*Geobacillus thermoleovorans* sub.sp. *stromboliensis*	23.2	*M*: 0.25 g L^−1^; *C*_0_: 200 mg L^−1^; *t*: 15 min; pH: 5	[42]
*Bacillus* sp.	43.5	*M*: 1 g L^−1^; *C*_0_: 300 mg L^−1^; *t*: 24 h; pH: 6-7	[43]
Sewage activated sludge	12.7	*M*: 1 g L^−1^; *C*_0_: 300 mg L^−1^; *t*: 24 h; pH: 6-7	[43]

## Data Availability

The data used for this manuscript are available from the corresponding author upon request.
